# Transcriptional activity and epigenetic regulation of transposable elements in the symbiotic fungus *Rhizophagus irregularis*

**DOI:** 10.1101/gr.275752.121

**Published:** 2021-12

**Authors:** Alexandra Dallaire, Bethan F. Manley, Maya Wilkens, Iliana Bista, Clement Quan, Edouard Evangelisti, Charles R. Bradshaw, Navin B. Ramakrishna, Sebastian Schornack, Falk Butter, Uta Paszkowski, Eric A. Miska

**Affiliations:** 1 Wellcome Trust/Cancer Research UK Gurdon Institute, University of Cambridge, Cambridge CB2 1QN, United Kingdom;; 2 Department of Genetics, University of Cambridge, Cambridge CB2 3EH, United Kingdom;; 3 Tree of Life, Wellcome Sanger Institute, Cambridge CB10 1SA, United Kingdom;; 4 Quantitative Proteomics, Institute of Molecular Biology, 55128 Mainz, Germany;; 5 Sainsbury Laboratory, University of Cambridge, Cambridge CB2 1LR, United Kingdom;; 6 Crop Science Centre, University of Cambridge, Cambridge CB3 0LE, United Kingdom

## Abstract

Arbuscular mycorrhizal (AM) fungi form mutualistic relationships with most land plant species. AM fungi have long been considered as ancient asexuals. Long-term clonal evolution would be remarkable for a eukaryotic lineage and suggests the importance of alternative mechanisms to promote genetic variability facilitating adaptation. Here, we assessed the potential of transposable elements for generating such genomic diversity. The dynamic expression of TEs during *Rhizophagus irregularis* spore development suggests ongoing TE activity. We find *Mutator*-like elements located near genes belonging to highly expanded gene families. Whole-genome epigenomic profiling of *R. irregularis* provides direct evidence of DNA methylation and small RNA production occurring at TE loci. Our results support a model in which TE activity shapes the genome, while DNA methylation and small RNA–mediated silencing keep their overproliferation in check. We propose that a well-controlled TE activity directly contributes to genome evolution in AM fungi.

The arbuscular mycorrhizal (AM) symbiosis is hundreds of millions of years old and a majority of the world's plant species are hosts to AM fungi (AMF) (Lutzoni et al. 2018). As such, these fungi exist in a wide range of environments and can even engage in symbioses with multiple plant species simultaneously. The complex life cycles of AMF suggest a requirement for strong developmental and phenotypic plasticity. However, although AMF carry meiosis-related genes (Halary et al. 2011) and heterokaryotic strains originating from two parental strains have been described (Ropars et al. 2016), genetically distinct strains as a product of meiosis have never been reported and direct evidence of sexual reproduction is lacking (den Bakker et al. 2010; Chen et al. 2018b; Reinhardt et al. 2021). This has led to the hypothesis that AMF are ancient asexual organisms, which raises a key question on how these fungi were able to diversify their gene inventory and fill such varied ecological niches.

Genome assemblies are available for a number of AMF species, including the model *Rhizophagus irregularis* (Tisserant et al. 2013; Chen et al. 2018a; Maeda et al. 2018). Genomic analyses of AMF have revealed contents of repetitive sequences ranging from 23% to 43% (Chen et al. 2018a; Morin et al. 2019). These repeats consist of transposable elements (TEs) and expanded gene families that occasionally form tandemly repeated arrays of duplicate genes (Tisserant et al. 2013; Chen et al. 2018a; Maeda et al. 2018; Morin et al. 2019; Silvestri et al. 2019, 2020). Expanded family genes are either orphans (no significant homolog can be identified) or contain protein domains related to signaling and RNA interference (RNAi), such as kinase domains, BTB/POZ (Broad-Complex, Tramtrack and Bric-a-brac/Poxvirus and Zinc finger) domains, Sel1-like tetratricopeptide repeats, and Kelch-like and P-element Induced WImpy testis (PIWI) domains. These high copy number genes form strain-specific accessory gene sets and have been proposed to play roles in perception and interaction with the environment (Reinhardt et al. 2021).

Transposons are repetitive DNA sequences that colonize genomes and generate intra- and inter-specific genetic variability. By moving and replicating within genomes, TEs cause chromosomal rearrangements and compartmentalization, deletions, duplications, and regulatory changes (Chuong et al. 2017). As their roles can be adaptive as well as deleterious, eukaryotes have developed defense mechanisms to control their proliferation. Three mechanisms of defense have been described in fungi: repeat-induced point mutation (RIP) (Gladyshev 2017), DNA cytosine methylation (Bewick et al. 2019), and RNAi (Torres-Martínez and Ruiz-Vázquez 2017). Signatures of RIP have not been detected in the AMF *Gigaspora margarita* or in species of the Mucoromycotina, a sister subphylum to the Glomeromycotina to which AMF belong (Venice et al. 2020). However, the presence of DNA cytosine methyltransferases and RNAi pathway genes encoded in AM genomes suggests their role in TE silencing.

The genomic organization of TEs in AMF is still largely unknown, as are the mechanisms that keep them under control. In this study, we investigate the organization of TEs, genes, DNA methylation, and RNAi on a global genome level in the model AMF *R. irregularis*.

## Results

### The TE landscape of *R. irregularis*

**Figure 1.**
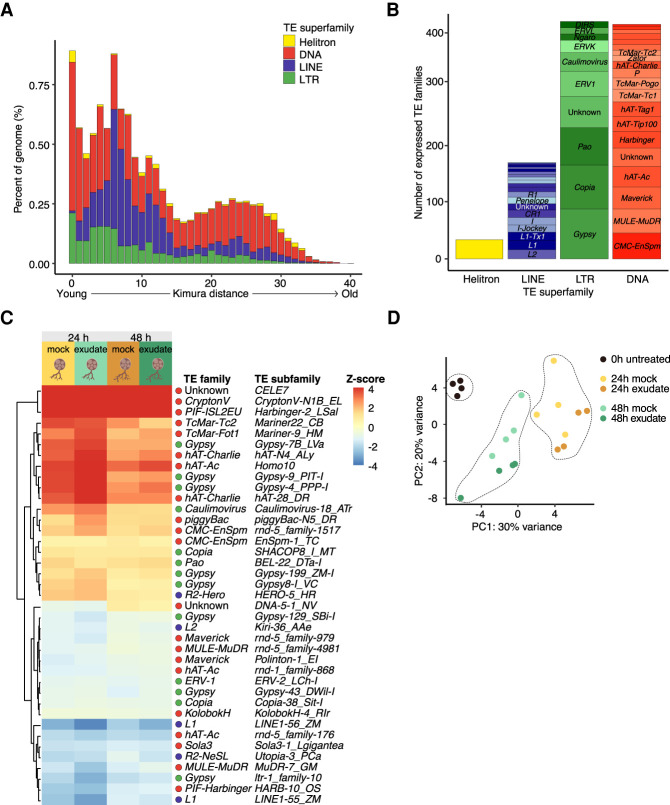
Transposon expression during *R. irregularis* spore development. (*A*) Repeat landscapes showing Kimura distance-based copy divergence analysis of TEs in *R. irregularis* genome shown as genome coverage (%) for each TE superfamily plotted against Kimura distance. Clustering was performed according to their Kimura distances of TEs (CpG adjusted K-value from 0 to 50). TE copies with a low Kimura distance value have a low divergence from the consensus sequence and may correspond to recent replication events. Sequences with a higher Kimura distance value corresponded to older divergence. Note that we omitted unclassified elements. (For repeat landscape including unclassified elements, see Supplemental Fig. S1.) (*B*) Number of expressed transposon subfamilies, grouped superfamilies. (*C*) Heat map and hierarchical clustering of differentially expressed TE subfamilies (|log_2_FC| > 0.5; FDR < 0.05) in the spore developmental assay. Five conditions were used in total, a 0-h control treatment, 24-h mock and rice exudate treatments, and 48-h mock and rice exudate treatments (four replicates per treatment). Expression of 24-h and 48-h conditions were normalized against expression in the control, 0-h condition. (*D*) Principal component analysis of TE subfamily expression across all replicates and conditions.

In order to characterize the content and distribution of TEs in the genome of *R. irregularis*, we generated a new TE annotation of the published *R. irregularis* genome (Maeda et al. 2018). Our annotation revealed a 47% repeat coverage, which is consistent with the previous report by Maeda et al. (2018) (Supplemental Fig. S1A,B). Of these repeats, 12% could be classified into TE families, which is within the range of what is typically observed in fungi (0.2%–30%) (Castanera et al. 2016) but lower than that observed for the obligate biotroph pathogen *Blumeria graminis* (∼45%) (Spanu et al. 2010). The genome of *R. irregularis* contains many DNA transposons and retrotransposons such as *Gypsy* LTRs, LINE *R1*, *Hobo*, and *Tc1* (Supplemental Fig. S1B). A portion of repetitive sequences occupying 32% of the genome could not be classified into TE families (Supplemental Fig. S1A–C). Sequences of high copy number (kinase, *BTB/POZ*, *Sel1*-like, *Kelch*-like) and orphan genes are inherently repetitive and therefore account for some of these unclassified repeats (8% of the genome) (Supplemental Fig. S1A). The remaining unclassified repeats (occupying 24% of the genome) remain to be characterized. Subsequent analyses were focused on classified TEs. Using divergence analysis based on calculated Kimura distance, we observe two waves of transposon expansion (Fig. 1A). The most recent expansions mostly consist of DNA transposons and retrotransposons (Kimura distance 0–1) (Fig. 1A), more specifically *Maverick*, *CMC*, *hAT*, *MULE*, and *Gypsy* elements (Supplemental Fig. S1C). These TE families may have therefore contributed to genome architecture in *R. irregularis*.

### Expression of TEs in developing *R. irregularis* spores

The process leading to TE activation is not well understood, but transcription is a precondition for their mobility. We measured the expression levels of TE transcripts in a developmental assay where *R. irregularis* spores were exposed to either medium containing rice root-derived exudates or a mock nutrient medium. Spore response to these two conditions was examined at three time points: a 0-h untreated time point, and 24 h and 48 h posttreatment. An initial analysis was carried out to establish the full scope of TE and gene expression at any of these time points. Using tools optimized for quantifying highly repetitive sequences, we detected significant expression for members of all TE superfamilies (Fig. 1B). The most represented families included *Gypsy*, *Copia*, *Pao*, *CMC-EnSpm*, and *MULE-MuDR* elements (Fig. 1B). We then investigated the expression of TEs at the subfamily level in the spore development assay. Although a majority of differentially expressed TE families were DNA transposons, LINEs and LTRs also displayed both up- and down-regulation (Fig. 1C; Supplemental Table S1). Principal component analysis (PCA) of TE subfamily expression showed that biological replicates formed discrete clusters based on time point but did not cluster well in response to treatment (rice exudates or mock treatment) (Fig. 1D). This may indicate that TE subfamily expression dynamics are not dependent on plant-derived compounds under these conditions. We next sought to analyze TE expression with locus-level resolution. We detected 786 individual TEs that overlapped with expressed genes (Supplemental Table S2). Of these, 232 had significant expression and shorter lengths (Supplemental Fig. S2A) but were excluded from following analyses as their expression could be attributed to expression of the genes they reside in. Two thousand thirty nongenic TEs displayed wide ranges of expression levels, belonged to all TE superfamilies (Supplemental Fig. S2B), and could be categorized into evolutionary divergence bins between 0 and 40 (Supplemental Fig. S2C), suggesting that TEs of all ages are transcribed. Dynamic expression of TEs suggests ongoing activity; however, in order to mobilize, TEs would likely need to be full-length elements. We therefore examined the length and relative age of expressed TEs. TEs with lengths of over 2 kb and low Kimura distance were detected (Supplemental Fig. S2D, shaded area). However, due to ambiguous mapping of reads to individual TE copies, we could not validate the expression of full-length copies. Taken together, these data show fluctuations in TE subfamily expression in developing spores, which may indicate relaxation of TE silencing during spore development and suggests a potential ongoing TE activity in *R. irregularis*.

### Methylome analysis of spore DNA using single-molecule sequencing

**Figure 2.**
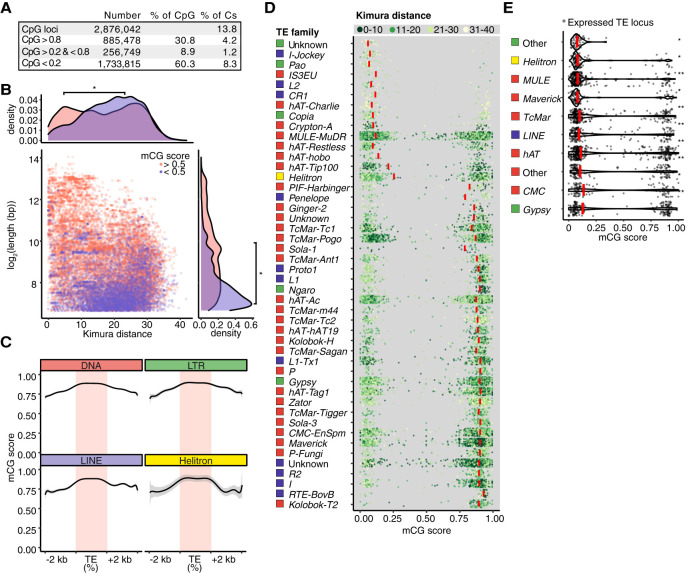
Interplay between transposons and DNA methylation in *R. irregularis* spores. (*A*) Absolute and relative proportions of mCG sites in *R. irregularis* spores. (*B*) Length of TE loci relative to divergence expressed as Kimura distance. Point color indicates high and low mCG score (pink and purple, respectively). Density plots depict TE length and divergence for elements belonging to each mCG score category. A Kruskal–Wallis H test was performed to compare the mCG score distributions of lowly and highly methylated TEs. (*) *P*-value < 2.2 × 10^−16^. (*C*) Metagene plots displaying mCG levels across the body, 2-kb upstream and downstream sequences of TE copies belonging to four TE superfamilies. (*D*) Average methylation (mCG score) of individual TE loci (length >100 bp and copy number >20), grouped into TE families. Red bars show the median values and point color indicates the relative age of each TE expressed as Kimura distance and grouped into bins. (*E*) mCG scores of expressed TEs. Red bars represent the median values of each superfamily. Significance was assessed by a Kruskal–Wallis H test comparing the mCG score distribution of expressed TEs to the mCG score distribution of nonexpressed TEs of the same class. (*) Kruskal–Wallis *P*-value < 1 × 10^−36^, > 1 × 10^−100^. (**) Kruskal–Wallis *P*-value < 1 × 10^−100^.

Detection of TE expression led us to hypothesize that epigenetic mechanisms may be targeting TEs in spores. Cytosine methylation is an important factor in suppression of TE transcription (Zemach et al. 2010). We therefore assessed its role in regulating TEs by surveying genome-wide 5-methylcytosine (5mC) using Nanopore long-read direct sequencing of DNA extracted from untreated spores. A total of 2,876,042 mCG sites were identified, accounting for 13.8% of total genomic cytosine content. CG site methylation status displayed a strong bimodal distribution, with sites either highly (30.8% of CpGs >0.8) or weakly (60.3% of CpGs <0.2) methylated (Fig. 2A). This trend of bimodal CG site methylation is also observed in plants, animals, and other fungi (Zhang et al. 2006; Elango et al. 2009; Wang et al. 2013; Montanini et al. 2014). In fungi studied so far, methylation levels are highest in repeats and transposons (Bewick et al. 2019). To examine whether this was the case in *R. irregularis*, we profiled the levels of mCG in classified TEs. In general, short and evolutionarily older TE loci displayed low mCG scores (Fig. 2B), suggesting a loss of mCG in old, degenerated TEs, a phenomenon also observed in inactive rice retrotransposons and mammalian LINEs (Vonholdt et al. 2012; Castro-Diaz et al. 2014). We then categorized TE copies into their respective superfamilies and examined mCG levels along the length of TEs and in flanking regions. We found higher mCG levels within the TE locus than in the immediate upstream and downstream regions (Fig. 2C). Although median mCG levels were high in most transposon families, recently expanded *TcMar*, *hAT*, *Gypsy*, and *Maverick* displayed numerous copies with low methylation (Fig. 2D). *MULE-MuDR* elements found in our analysis to be expressed and down-regulated during spore development (Fig. 1C) also displayed a lower than average median mCG score (12.5%) (Fig. 2D). If occurring in the absence of suppression through other control layers such as CHG/CHH methylation, histone modification, or RNAi, DNA hypomethylation can lead to TE derepression. We therefore examined whether low mCG levels could be linked to TE loci expression. Indeed, members of all families of expressed TEs were significantly associated with lower mCG levels compared to their respective nonexpressed counterparts (Fig. 2E). These data show that *R. irregularis* TEs are generally highly methylated, but older, short, and expressed TEs tend to have lower mCG levels. DNA methylation may therefore be associated with the regulation of TEs during development and over evolutionary time.

### High copy number genes are located next to *Mutator*-like elements (*MULE*s)

**Figure 3.**
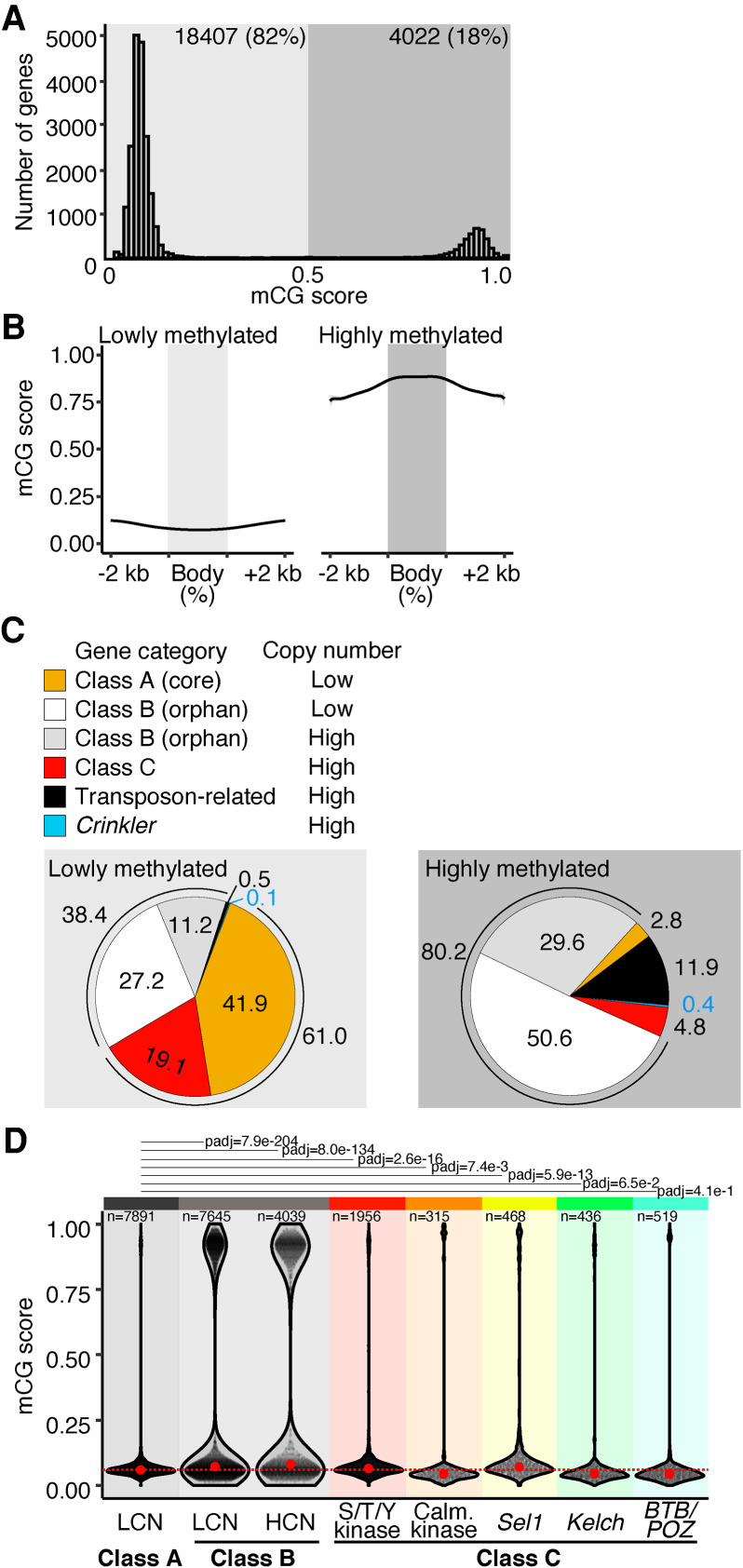
Methylation state of genes. (*A*) Distribution of methylation levels of *R. irregularis* genes. (*B*) Metagene plots of mCG methylation across genes with low (*left*) or high (*right*) mCG scores and their 2-kb upstream and downstream sequences. (*C*) Protein domain predictions of lowly and highly methylated genes. Pie charts show percentage of genes of each class, as categorized by the identity of their protein domains. Core (Class A) are nonrepeated genes with an identifiable protein domain. Class B genes contain no known protein domain. The class C gene category includes serine/threonine/tyrosine (S/T/Y) kinase, calmodulin-dependent kinase, *Sel1*-like, *Kelch*-like, and *BTB/POZ.* Transposon-related genes contain domains such as reverse transcriptase. Crinkler-type genes have a crinkler domain. (*D*) mCG score distribution of Class A (low copy number; LCN), Class B genes (high/low copy number; HCN/LCN), and high copy number Class C genes (rainbow-colored). A Kruskal–Wallis Dunn's multiple comparisons test (Benjamini–Hochberg correction) comparing mCG score distributions of gene groups was used to assess significance. Only adjusted *P*-values for comparisons to Class A are shown. The dotted red line highlights Class A median mCG levels.

As the genome of *R. irregularis* has a high content of repetitive genes (Supplemental Fig. S1A), we hypothesized that repetitiveness may correlate with transcriptional silencing by DNA methylation. We profiled mCG levels in gene bodies and observed that, although the majority of genes displayed very low mCG levels, 18% of all *R. irregularis* genes are highly methylated (Fig. 3A). This feature sets *R. irregularis* apart from other fungi, which have so far been shown to have low gene body methylation (Bewick et al. 2019). The distribution of mCG along the sequence of highly methylated genes was similar to that seen in TEs (Fig. 3B). We then examined the functional annotation of lowly and highly methylated genes and categorized them based on predicted function and repetitiveness: (1) Class A are core, low copy number (LCN) genes; (2) Class B have no known protein domain (orphan genes) and were further classified into low and high copy number groups; and (3) Class C are high copy number (HCN) signaling-related genes with serine/threonine/tyrosine kinase, calmodulin-dependent kinase, BTB/POZ, Sel1-like, or Kelch-like domains (Fig. 3C). Only the most highly amplified gene families with known proteins domains (described in Tisserant et al. 2013; Maeda et al. 2018) were included in the HCN Class C. The remainder are either genes with transposon-related domains (e.g., reverse transcriptase) or crinkler domains (Pfam PF20147), part of a subfamily of candidate AMF secreted effector proteins (Voß et al. 2018). In plant pathogenic fungi, such candidate secreted effector genes tend to be located in TE-rich genomic islands (Faino et al. 2016), and some have co-evolved with particular TEs (Sacristán et al. 2009; Fouché et al. 2018). We found that 61% of lowly methylated genes were core or HCN Class C genes (Classes A and C) (Fig. 3C, left panel. Core Class A and Class C genes therefore tend not to be found in highly methylated regions, consistent with their presence in genomic compartments that are permissive to transcription. Most highly methylated genes had no known protein domain (Class B orphans, 80.2%) (Fig. 3C right panel) or had a transposon (11.9%), signaling (Class C, 4.8%), or crinkler (0.4%) domain. A proportion of both high and low copy number Class B orphan genes was found to be highly methylated (Fig. 3C), suggesting that repetitiveness is not necessarily associated with a high methylation status.

We then compared the methylation score distributions of Class A, B, and C genes. Class B contained the highest number of genes with high methylation scores, with both LCN and HCN groups displaying a bimodal distribution similar to that of transposons (Figs. 2D, 3D). Serine/threonine/tyrosine kinase and *Sel1*-like gene families in particular displayed significantly higher average mCG scores than Class A, whereas other Class C subgroups had lower mCG scores (Fig. 3D). Overall, this data indicates that subsets of Class C and both LCN and HCN Class B genes are found in highly methylated regions, whereas core genes are not. Differences in the mCG context of gene classes resembles the way some pathogenic fungi genetically compartmentalize their effector genes in TE-rich regions, displaying a so-called two-speed genome (Faino et al. 2016). Looking at gene density, we could not find evidence that *R. irregularis* carries a two-speed genome (Supplemental Fig. S3A). However, we observed that Class A genes tend to harbor shorter intergenic distances, compared to Class B and C genes (Supplemental Fig. S3B–D). HCN and orphan genes are therefore sparse and perhaps nested in repeat-rich regions.

**Figure 4.**
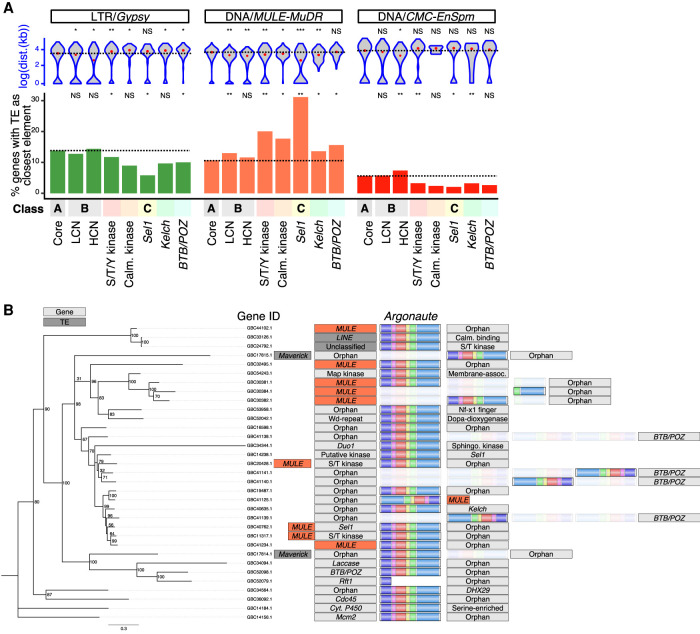
Location of genes relative to TEs. (*A*) Identity and distance of TEs closest to genes of classes A, B, and C. *Top*: log_10_-transformed distance (bp) between genes and closest TE of the displayed classes (Class A, B, and C genes, and LTR/*Gypsy*, DNA/*MULE-MuDR*, or DNA/*CMC-EnSpm* TEs). Class B genes are split into low and high copy number categories (LCN and HCN). Black dashed line highlights the median value of Class A. Significance was assessed via a Kruskal–Wallis H test comparing the distance distribution of each TE to repeated gene groups to Class A genes. (*) *P*-value <0.05, > 0.001. (**) *P*-value < 0.001. *Bottom*: proportion of genes of each class with a *Gypsy*, *MULE-MuDR*, or *CMC-EnSpm* element as their closest TE. Underrepresentation or enrichment significance of Class B and C genes was assessed by a Fisher's exact *t*-test comparing the occurrences of each TE class closest to genes of each family, compared to Class A core genes. (*B*) DNA-based phylogenetic tree and representation of the genomic context surrounding *AGO*genes. Bootstrap values (100 replicates) are indicated. The class of closest genes and/or transposable elements are shown. MULEs are colored in red, repeat elements are dark gray, and genes are light gray. Colored regions on *AGO*genes represent the six typical protein domains: N-terminal (purple), linker 1 (pink), PAZ (red), linker 2 (yellow), MID (green), and PIWI (blue).

Supporting this observation, HCN signaling-related genes (Class C) were previously reported to be localized near TEs (Maeda et al. 2018). As we found subsets of Class B and C genes to be highly methylated and located in gene-sparse regions, we hypothesized that expansions of these gene classes could have been caused by TEs. We first examined which TE families were most often close to genes (Supplemental Table S3). *MULE*s were the most represented, consistent with their known bias for inserting near genes (Cresse et al. 1995; Lisch 2015). For the top three TE families found near genes, we quantified the frequency at which each element could be found next to members of each gene category and the distance between them (Fig. 4A, bottom and top panel, respectively). We found that LCN and HCN Class B genes tend to be located significantly closer to *Gypsy*, *MULE*, and *CMC-EnSpm* elements than Class A genes (Fig. 4A, top panel) but are not necessarily significantly enriched (Fig. 4A, bottom panel). Orphan gene copy number is therefore not necessarily associated with proximity to the TEs analyzed here. *Gypsy*and *CMC-EnSpm*elements were significantly underrepresented next to most subgroups of Class C genes, compared to Class A. They were either further away or located at a distance that was not significantly different from this element's distance from Class A genes. *MULE*s, however, were the most represented TE family found proximal to all subgroups of Class C genes and, except for *BTB/POZ*, they were closer to Class C genes than they were to Class A genes. Thus, Class C genes are overrepresented next to *MULE*s specifically, which suggests a link between *MULE* activity and expansion of these signaling-related genes. This has been observed in previous studies of plant genomes in which *MULE*s are particularly active (Cresse et al. 1995; Lisch 2015). In rice, *MULE*s carrying gene fragments or entire genes, called *Pack-MULE*s, have duplicated sequences of over 1500 genes (Ferguson et al. 2013). In maize, *MULE*s often contain receptor protein kinase and calmodulin insertions, similar to the phenomenon observed in *R. irregularis* (Fig. 4A; Tan et al. 2011). In addition, we found *MULE*s within close proximity of eight *AGO* genes, with five of these genes immediately next to *MULE*s (Fig. 4B). A phylogenetic analysis of *AGO* gene sequences indicated that *MULE-AGO* pairs tend to cluster into groups, suggesting that *MULE*-linked *AGO*s have expanded due to a cut-and-paste mechanism. AGO proteins associate with small RNAs (sRNAs) such as small interfering RNAs and microRNAs, and function in RNA-based silencing mechanisms. We propose that *MULE*s may have paradoxically caused the expansion of a pathway that is well-known to suppress TE activity in fungi, plants, and animals.

### A subset of *R. irregularis* small RNAs are 2′-*O*-methylated and Argonaute-loaded

As the proximity between *MULE*s and *AGO* genes suggests a role for TEs in expanding the RNAi gene repertoire, we wondered whether RNAi was involved in the regulation of TEs. RNAi typically relies on four core components: Dicer, Argonaute (AGO), sRNA 2′-*O*-methyltransferase (HEN1), and RNA-dependent RNA polymerase (RdRP) (Supplemental Fig. S4A). sRNAs are generally generated through the cleavage of double-stranded RNA precursors by Dicer proteins and are sorted into specific AGOs with different regulatory capacities (Chang et al. 2012). AGOs bind sRNAs and use them as guides to base-pair with RNA targets and trigger their repression (Hutvagner and Simard 2008). HEN1 is an RNA methyltransferase that 2′-*O*-methylates the 3′ end of sRNAs, protecting them from degradation by exonucleases and increasing their stability (Ji and Chen 2012). In eukaryotes, RdRPs are recruited to target RNAs, which they use as a template to produce complementary RNAs which trigger a secondary amplification mechanism to generate more sRNAs and enhance silencing activity (Chang et al. 2012). Examination of RNAi pathway genes in the genomes of mycorrhizal fungi revealed that AM species harbor *AGO* and *RdRP* gene expansions and have maintained the sRNA methyltransferase (*HEN1*) gene (Supplemental Fig. S4B; Tisserant et al. 2013; Silvestri et al. 2019, 2020). For RNAi pathways to be functional in spores, proteins involved in sRNA biogenesis and function must be expressed. We used label-free quantitative proteomics to profile global protein expression during spore development. We first removed any peptides that could be derived from rice root exudates or that matched to multiple species, leaving 3475 *R. irregularis* proteins for further analyses (Supplemental Fig. S4C). In all treatments examined, we detected unique peptides mapping to DCL, HEN1, 10 AGO proteins, three AGO-binding proteins (ARB1 and ARB2 in fission yeast) (Buker et al. 2007), and two genes involved in RNAi-mediated heterochromatin assembly (HRR1 and STC1 in fission yeast) (Motamedi et al. 2004; Bayne et al. 2010) but found no RDRP (Supplemental Fig. S4D; Supplemental Table S4). Most RNAi pathway protein components are detected and, because RDRPs are not always essential for sRNA biogenesis and function, it is reasonable to expect functional RNAi in spores. Detection of HRR1 and STC1 homologs suggests the existence of an RNAi-coupled chromatin modification pathway in *R. irregularis*, perhaps in addition to transcriptional or post-transcriptional RNA silencing. Levels of RNAi pathway proteins were relatively high compared to the proteome-wide distribution of label-free quantitation scores (Supplemental Fig. S4E). Proteins typically involved in sex and meiosis, as well as putative effectors, were detected (Supplemental Table S4). We then compared protein expression levels during the spore development assay. One hundred eleven proteins were differentially expressed in at least one condition compared to untreated controls (Supplemental Fig. S4F; Supplemental Table S5). One AGO protein (A0A2H5UB68) was significantly down-regulated at 48 h in both mock and exudate treatments, suggesting active regulation of this RNAi factor during spore development. PCA of protein expression indicated that biological replicates do not form discrete clusters based on treatment or time point (Supplemental Fig. S4G). This may indicate subtle protein expression dynamics under these conditions. Functional enrichment of differentially expressed proteins revealed significant GO terms in 24-h and 48-h exudate treatments that are, respectively, associated with glycine transport and DNA replication, suggesting active regulation of amino acid metabolism and replication in spores (Supplemental Fig. S4H).

**Figure 5.**
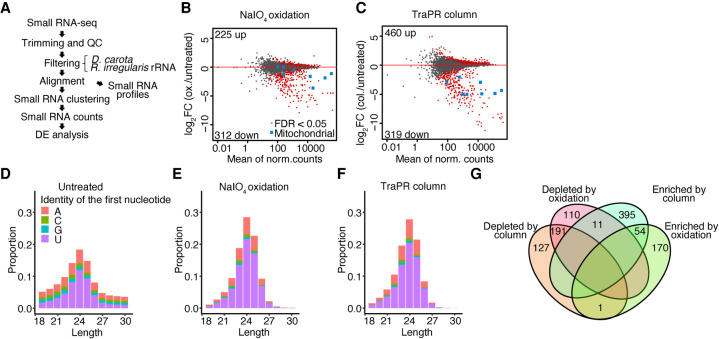
Isolation of 2′-*O*-methylated and Argonaute-loaded small RNA. (*A*) Schematic representation of the sRNA-seq analysis pipeline used in this study. (*B*,*C*) Plot of mean normalized counts against log_2_ fold change (log_2_FC) in sRNAs sequenced following enrichment using NaIO_4_ oxidation (*B*) or TraPR column purification (*C*), compared to sRNAs sequenced following no treatment. Points represent individual sRNA loci. Significantly differentially expressed loci are shown in red. Mitochondria-derived sRNA loci are shown in blue. (*D*–*F*) Length distribution and first nucleotide bias of the sRNAs in untreated (*D*), NaIO_4_-treated (*E*), and TraPR column-extracted (*F*) sRNA libraries. (*G*) Significantly differentially expressed sRNA loci. sRNAs enriched by NaIO_4_ or column-purification were induced compared to expression in untreated samples, and sRNAs depleted by NaIO_4_ or column-purification were down-regulated.

Expression of the sRNA methyltransferase HEN1 suggests that this enzyme could be actively modifying sRNAs in spores. We investigated the presence of 2′-*O*-methyl modifications of sRNA and profiled the full spectrum of functional AGO-sRNA complexes present in *R. irregularis* spores. We sequenced and compared the sRNA libraries sequenced from three RNA extraction methods: (1) total RNA extraction; (2) enrichment of 2′-*O*-methylated sRNAs using sodium periodate (NaIO_4_) oxidation (Yu et al. 2005); and (3) TraPR column-based isolation of AGO-loaded sRNAs (Fig. 5A; Grentzinger et al. 2020). We found that NaIO_4_ treatment and TraPR isolation produced similar sRNA profiles with a more well-defined peak than those observed using a total-RNA treatment (Fig. 5D–F). The NaIO_4_ and TrAPR sRNA profiles displayed a length distribution centered at 24 nucleotides and a strong bias for sequences beginning with a 5′-terminal uridine or adenine (Fig. 5E,F). In plants and animals, the identity of the 5′-terminal nucleotide often determines which AGO protein a sRNA is loaded into (Mi et al. 2008; Stein et al. 2019). The 5′U and 5′A biases observed here suggest an evolutionary pressure for sRNAs to begin with a specific nucleotide, perhaps driven by structural specialization of sRNA binding pockets of *R. irregularis* AGO proteins.

We then compared sRNA sequences produced using NaIO_4_ and TraPR column treatments. The clustering analysis of sRNA loci revealed that both methods deplete most mitochondria-derived reads. Mitochondria-derived sRNAs were highly abundant, sometimes representing over 10% of all reads. Of 3495 small RNA loci, 631 were significantly enriched by either column treatment, oxidation, or both (Fig. 5G). Among those, 54 sRNA loci were enriched by both treatments, pointing to a subset of sRNAs being both 2′-*O*-methylated and Argonaute-loaded. AGO-loaded sRNAs can be expected to be functional and, although the purpose of sRNA modification in AMF is unknown, the 2′-*O*-methylated group likely represents a pool of highly stable sRNAs. sRNA loci significantly depleted or not enriched by either method were removed, leaving a total of 3067 sRNA loci for further analyses.

**Figure 6.**
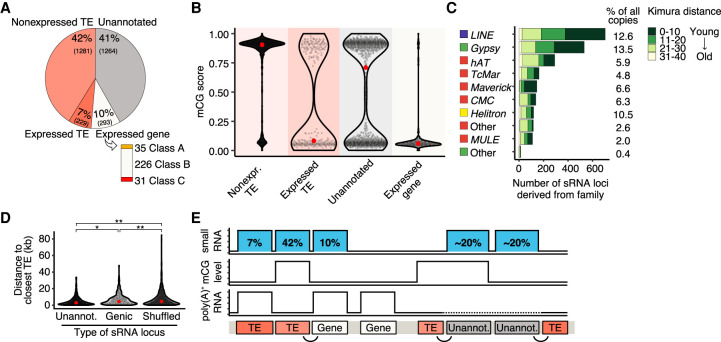
Genomic origin of small RNA–producing loci. (*A*) Genomic location of 3067 *R. irregularis* sRNA loci. The number of sRNA loci derived from nonexpressed and expressed TEs, unannotated regions, and genes is represented in a pie chart. Bar chart shows the number of genes of each class that produce sRNA. (*B*) mCG scores of expressed sRNA loci derived from nonexpressed TEs, expressed TEs, unannotated features, and expressed genes. Red dots represent the median mCG values of sRNAs from each feature. (*C*) Number of sRNA loci associated with different TE superfamilies. Percentage of TE copies from each superfamily that produce sRNA is shown on the *right*. Green color code indicates the relative age of sRNA-producing TEs, represented in Kimura distance bins between 0 and 40. Expressed sRNAs have been grouped into bins based on Kimura distance and hence relative age, represented using color coding. (*D*) Distance from sRNA loci derived from unannotated regions and expressed genes to closest known TE. Significance was assessed by a nonparametric Kruskal–Wallis test comparing the distance distribution of unannotated or genic sRNA loci to shuffled loci, respectively. A Kruskal–Wallis H test comparing unannotated and genic sRNA loci to shuffled loci was used to assess significance. (*) *P* = 2.8 × 10^−6^, (**) *P* < 2.2 × 10^−16^. (*E*) Schematic diagram of the genomic distribution of sRNA loci hypothesized due to data from this study of the *R. irregularis* genome, sRNAome, methylome, and transcriptome. sRNA loci, methylation levels, and RNA expression are displayed. The proportion of sRNA loci corresponding to each genomic context is indicated in blue boxes. Curvy lines highlight the proximity between TEs and genic and unannotated sRNA loci. Expression of unannotated regions was not analyzed and is represented by a dotted line.

To define the genomic origin of sRNA loci, we searched for overlap with genomic features. We found 1510 (49%) sRNA loci matching classified TE sequences, of which 7% showed significant RNA expression (Fig. 6A). Of the remaining sRNA loci, 41% were derived from unannotated regions (no gene, no classified TE) and 10% originated from expressed protein-coding genes. No sRNA locus was derived from nonexpressed protein-coding genes. The majority of genes that produced sRNA had no known protein domain (Class B). sRNA loci derived from nonexpressed TEs were, in general, highly methylated, whereas sRNA loci derived from expressed TEs had lower methylation scores (Fig. 6B). sRNA loci originating from unannotated regions displayed a bimodal distribution similar to that of TEs and Class B genes (see Figs. 2B, 3D). Lastly, coding genes producing sRNAs were mostly nonmethylated, consistent with their expression. All TE superfamilies (DNA, LTR, LINE, and Helitron) produced sRNA (Fig. 6C), and most loci were derived from LINE and *Gypsy*retrotransposons, with, respectively, 12.6% and 13.5% of elements of each family producing sRNA. sRNA-targeted TEs tended to have a lower divergence (Fig. 6C), suggesting a role for sRNA in scanning recently active transposons. The production of 24-nt long sRNA from young TE loci is also observed in plants, where 24-nt mobile sRNAs direct DNA methylation to silence active elements (Hollister et al. 2011; Gong et al. 2015; Bousios et al. 2016).

We next asked what could explain the expression of sRNA from non-TE regions. We observed that sRNA loci derived from unannotated regions and genes were located significantly closer to TEs than a set of simulated sRNA loci shuffled randomly throughout the genome (Fig. 6D). Although we do not know what the function of these loci are or why these specific genes produce sRNA, their proximity to TEs suggests a link with TE activity or regulation. Together, our data show that most sRNAs are produced from TEs (49%), unannotated regions (∼20%), or genes in the vicinity of TEs (∼20%) (Fig. 6E).

## Discussion

### Signs of recent or ongoing TE activity in *R. irregularis*

The genome of *R. irregularis* is highly repetitive and harbors many copies of evolutionary young TEs with low sequence divergence. Over 1000 TE families are expressed and 39 subfamilies (e.g., *MULE-MuDR*and *Gypsy*) are differentially expressed during spore development. Transcriptional activity is a crucial step toward transposition and may correlate with epigenetic changes. We provide two lines of evidence of epigenetic regulation occurring in *R. irregularis*: (1) high CG methylation levels at the majority of, but not all TE loci; and (2) sRNA production, particularly from evolutionarily young TE loci. Consistent with the activity of epigenetic pathways in spores, we detect protein expression of a DNMT1-related DNA methyltransferase (A0A2H5UGI7) (Supplemental Table S4) and 17 proteins typically involved in RNAi (Supplemental Fig. S4D). In AMF, defense against TEs therefore likely involves DNA methylation and RNAi.

### Uneven distribution of core and noncore genes may underlie their regulation

Most genes of AMF have no known protein domain, hence are of unknown origin and function or belong to highly expanded families such as kinases. We now find that *R. irregularis* genes are not evenly distributed throughout the genome: Core and noncore genes are, respectively, dense and sparse. Alongside this phenomenon, we find enrichment of high mCG methylation in noncore orphan, kinase, and *Sel1*-like genes. In contrast, conserved genes including those thought to be involved in phosphate transport and metabolism tend to harbor 6mA methylation marks (Chaturvedi et al. 2021). Taken together, these data indicate that the genomic location of different gene classes may be linked with layers of epigenetic control. Recent chromatin conformation capture experiments confirmed the existence of two genomic compartments in *R. irregularis*, one enriched with core genes and the other with noncore genes and repeats (Yildirir et al. 2021). The location of noncore genes in regions with dynamic epigenetic regulation and different evolutionary speeds may underlie the adaption of AMF to hosts and environments.

### Proximity between *MULE*s and high copy number genes suggests transposon-mediated expansions

Genome analyses of five *R. irregularis* strains revealed that roughly half of their gene repertoires is not shared, raising questions about the evolutionary origin and biological function of accessory genes (for review, see Reinhardt et al. 2021). As we find these genes to be located in gene-sparse regions that are likely repetitive, copy number variations could be explained by a highly variable, rapidly mutating genomic compartment. Consistent with this idea, we found a link between the location of accessory gene classes and a specific transposon family, *MULE*. We therefore propose that expansion of signaling-related genes in *R. irregularis* may have been caused by *MULE*s, which could explain the presence/absence variation observed in different *R. irregularis* strains. Expansion of *AGO* genes also appears to be linked to *MULE*s, suggesting that RNAi pathway evolution may have been shaped by TE activity.

### Adaptation and evolution driven by TE activity

Perhaps the most intriguing feature of AMF is their thus far undocumented sexual reproduction. Although there appears to be potential for them to reproduce sexually (Halary et al. 2011; Ropars et al. 2016; Corradi and Brachmann 2017; Kokkoris et al. 2020), meiotic events have never been observed and genetically distinct strains created by meiotic divisions have never been identified. Nevertheless, sexual or parasexual reproduction could exist in AMF, though these events may occur rarely or in environmental conditions that are currently unknown. In the absence or rare occurrence of sex, or perhaps in parallel to sex, three mechanisms could generate genetic variation: (1) horizontal gene transfer, which is known to occur in AMF (Torres-Cortés et al. 2015; Li et al. 2018); (2) TE activity; and (3) cryptic recombination, the latter two of which were proposed to occur in AMF (Yildirir et al. 2020; Reinhardt et al. 2021). One could predict that these mechanisms bear more important roles in genome evolution of asexual or rarely sexual species than sexual species. TE activity generates significant adaptive genetic variation and has been shown to play roles in the evolution of genes encoding proteins involved in host interaction (Croll and McDonald 2012). Because orphan and accessory genes constitute the majority of *R. irregularis* genes, an important question arises: Do these genes have roles in plant-AMF interactions? Are they fast-evolving genes in the process of acquiring new function? We propose that among all expanded genes, AGOs may provide a crucial evolutionary advantage, by controlling TE activity. As *R. irregularis* sRNAs appear to target TEs and regions near them, we speculate that AGO expansions may help strike a balance in the evolutionary conflict between TE invasion and TE-driven adaptation.

As TE mobilization through a population is thought to be facilitated by sexual reproduction of their hosts, can TEs be strong drivers of genome evolution in asexual organisms or organisms with infrequent sexual reproduction? Bdelloid rotifers are asexual animals that display features similar to those observed in *R. irregularis*: (1) recent and ongoing TE activity; (2) large expansions of RNAi pathway genes (∼22 *AGO*, four *DCL*, and 37 *RdRP*) (Nowell et al. 2021); and (3) targeting of TEs by sRNA (Rodriguez and Arkhipova 2016). Although the cause of rotifer RNAi gene expansions is unknown, this research provides a case study of concomitant TE activity and expansion of RNAi genes in an asexual species. Without sex and recombination, organisms have limited ways of defending their genomes against unchecked TE expansions. As Nowell et al. (2021) propose, RNAi may have been required for ancient transitions from sexuality to asexuality. Thus, a controlled balance between TE activity and silencing may drive the adaptation and ecological success of AMF.

## Methods

### Production of rice exudates

Rice cultivar Nipponbare (*Oryza sativa* subsp. *japonica*) seeds were manually dehusked and sterilized by incubating with 3% sodium hypochlorite for 30 min on a platform shaker, followed by rinsing three times with diH_2_O. Sterile seeds were subsequently pregerminated on 0.7% Bacto agar plates for 4 d at 30°C. Seedlings were then transferred to trays of autoclaved sand and grown for 6 wk in a growth chamber under a 12-h light/12-h dark cycle at a 28°C daytime/23°C nighttime temperature and 60% humidity. From 2 wk onward, rice plants were provided twice a week with half-strength Hoagland's solution (25 µM phosphate) containing 0.01% (w/v) Sequestrine Rapid (Syngenta). For exudate collection, 6-wk-old plants were removed from soil, rinsed, and placed into 250-mL conical flasks containing 200 mL half-strength Hoagland's solution, with the roots submerged. Three rice plants were placed into each flask, which were then incubated for 3 d at the previously described plant growth conditions on a mechanical shaker at 50 shakes/min. Following the 3-d incubation, the half-strength Hoagland's solution containing plant exudates was filter-sterilized using 0.2-µM filters. Half-strength Hoagland's solution was prepared for use as a control treatment and was incubated without the addition of rice plants for 3 d at plant growth conditions before subsequent filter-sterilization.

### In vitro spore development assay

*R. irregularis* DAOM197198 grade A spores (Agronutrition) were suspended in 1× M Media at a concentration of 10,000 spores/mL. For a total of 20 samples, 5 mL (50,000 spores/sample) of the spore solution were aliquoted to individual 16.8-mL tissue culture wells, and the samples were incubated for 7 d at 30°C and 2% CO_2_. Following incubation, spore samples were either immediately frozen in liquid nitrogen for a 0 h time point or were re-incubated with either half-strength Hoagland's solution or with sterilized rice exudates, for 24 h or 48 h hours. Four spore samples were produced per treatment. Prior to freezing, all spore samples were drained using 40-µM cell strainers. For use in downstream processing, liquid nitrogen-frozen *R. irregularis* spore samples were homogenized using a mixer mill MM 400 and 25-mL grinding jars (Retsch), shaking at 25 shakes/sec for 20 sec.

### High-molecular-weight DNA extraction and sequencing

One hundred milligrams of ground spore material was resuspended in lysis buffer and processed as indicated in the protocol from Schwessinger and McDonald (2017) (version 4). Two successive rounds of cleanup were performed using a 0.4× volume of Ampure XP beads in DNA LoBind tubes following the manufacturer's protocol. DNA was eluted in 50 µL of 10 mM Tris, pH 8. DNA quality was assessed by running on a 0.5% agarose gel. Sequencing libraries were prepared using the Oxford Nanopore Rapid DNA sequencing kit SQK-RAD004 and sequenced on MinION flow cells FLO-MIN106D.

### RNA extraction, library preparation, and small RNA treatments

RNA extraction using an RNeasy Plant kit (Qiagen) was carried out on a portion of each ground spore sample. RNA integrity was assessed using an Agilent 2100 Bioanalyzer and RNA 6000 Pico kit and a Tapestation (Agilent). Paired-end poly(A)^+^ RNA libraries were produced and sequenced by Novogene UK Co., Ltd. with read lengths of 150 bp. For sRNA profiling experiments, 15–20 mg of ground spore samples were split into three equal volumes and subjected to one of three treatments: NaIO_4_ oxidation, TRaPR column treatment; and respective untreated conditions. For the oxidation treatment, 5 µL of 200 mM NaIO_4_ were added to 500 ng total RNA diluted in 24.5 µL of 1× borate buffer. Oxidation was performed at room temperature for 10 min, then RNA was precipitated at −20°C for 1 h with 0.1 V (4 µL) 3 M sodium acetate and 2.5 V(150 µL) ice cold 100% ethanol. RNA was centifuged at 13,000rpm at 4°C for 20 min, pellets were washed twice with 0.2 mL ice cold 80% ethanol, spun at 4°C for 5 min, air dried for 10 min, resuspended in 10.5 μL nuclease-free H_2_O, then ligated following the NEXTFLEX Small RNA-seq kit protocol. For Lexogen's TraPR Small RNA Isolation, 20 mg of ground spore material was suspended in TRaPR lysis buffer and the standard TRaPR experimental procedure and RNA extraction was carried out (Lexogen). The nontreated ground material of each sample was also suspended in TRaPR lysis buffer and RNA extracted through a phenol-chloroform extraction. All treated and RNA-extracted samples were then used to produce sRNA libraries using the NEXTFLEX Small RNA-seq kit v3 following the gel-based protocol, then sequenced on an Illumina HiSeq 1500 with read lengths of 50 bp. RNA and sRNA library sizes and statistics are presented in Supplemental Table S6.

### Proteomics

Twenty milligrams of each ground spore sample were resuspended in 1× LDS Buffer and 100 mM DTT and incubated at 70°C for 10 min. Proteins were separated on a 10% NuPage NOVEX Bis-Tris gel (Thermo Fisher Scientific) for 8 min at 180 V in 1× MES buffer (Thermo Fisher Scientific). The gels were fixated, stained with Coomassie Brilliant Blue G250 (Sigma-Aldrich), and afterwards destained with water. In-gel digestion and desalting on C18 StageTips were performed as previously described (Shevchenko et al. 2006; Rappsilber et al. 2007). LC-MS/MS analysis was carried out on an EASY-nLC 1000 system (Thermo Fisher Scientific) coupled to a Q Exactive Plus Orbitrap mass spectrometer (Thermo Fisher Scientific) via the nanoflex electrospray ion source. Peptides were separated on a 25-cm reversed-phase capillary with a 75-µm inner diameter packed in-house with Reprosil C18 resin (Dr. Maisch GmbH). The peptides were eluted during a 208-min gradient from 2% to 40% acetronitrile in 0.1% formic acid at a constant flow rate of 225 nL/min. The Q Exactive Plus was operated with a top 10 data-dependent acquisition method. For raw file peak extraction and the identification of protein groups, the MS raw files were searched with MaxQuant (version 1.6.10.43; 71) against the UniProt databases UP000236242 (*R. irregularis*) and UP000059680 (*O. sativa* subsp. *japonica*). The database searches were performed with MaxQuant standard settings using the label-free quantification (LFQ) algorithm (Cox and Mann 2008; Cox et al. 2014) and the match between runs option was activated. From the identified protein groups known contaminants, reverse entries, protein groups only identified by site or with no unique or less than two peptides were filtered out and excluded from the analysis. Missing LFQ values were imputed at the lower end of values within each sample and data plotted using the ggplot2 (https://ggplot2.tidyverse.org) and pheatmap packages in R (version 1.0.12, https://rdrr.io/cran/pheatmap/; R Core Team 2020). Functional enrichment analysis of differentially expressed proteins was performed using g:Profiler (Raudvere et al. 2019).

### Annotation of transposable elements

The *R. irregularis* DAOM197198 genome (Maeda et al. 2018) was used to perform de novo annotation using RepeatModeler2 (Flynn et al. 2020), which uses the RepeatScout (Price et al. 2005) and RECON (Bao and Eddy 2002) algorithms for TE discovery. We also used LTR_retriever (Ou and Jiang 2018) and LTR_harvest (Ellinghaus et al. 2008) to enhance detection of LTR retrotransposons. This combined new library was used to annotate the genome with RepeatMasker (Smit et al. 2013). The age of each TE copy was calculated as percentage of divergence (Kimura distance) from consensus models. Transposon families were binned according to divergence and plotted in relation to genome coverage (RepeatLandscape). To achieve retrieval of complete copies, the consensus sequences for the CMC-EnSpm, Crypton-A, and MULE families were manually curated by performing multiple rounds of BLAST (Camacho et al. 2009) alignments to retrieve the top 50 hits for each consensus, which were then extended, aligned with MUSCLE (Edgar 2004), and manually edited until the complete sequence of the consensus sequence was retrieved.

### Generation of transcriptome data for gene annotation

*R. irregularis* DAOM197198 spores were harvested from carrot root organ cultures under sterile conditions by dissolving the phytagel in 10 mM citrate buffer at pH 6.0. Carrot roots were carefully removed to avoid any contamination. Two-week-old *Nicotiana benthamiana* seedlings grown in vitro were transferred to autoclaved silver sand and inoculated with 3200 freshly extracted *R. irregularis* spores or water for mock controls. After 3 wk, plants were pulled out of sand and roots from four plants were pooled together per biological replicate for both mock and mycorrhized conditions. Sixty thousand spores were germinated and grown in liquid M-medium in the dark at 30°C supplemented with 2% CO_2_ for 7 d. Germinated spores were harvested using a 40-µm cell strainer (Sigma-Aldrich) and the excess of M-medium drained out before snap-freezing the samples in liquid nitrogen. All samples were produced in triplicate. Total RNA was extracted as previously described (Evangelisti et al. 2017). Complementary DNA (cDNA) libraries were prepared from 1 µg RNA using the TrueSeq RNA Sample Prep kit (Illumina). RNA and DNA libraries were quantified using a Qubit fluorometer (Thermo Fisher Scientific), and their integrity was checked on a TapeStation 2200 (Agilent) using RNA ScreenTape and High Sensitivity D1000 Screen Tapes. Libraries were diluted to 4 nM and sequenced on a NextSeq 500 Sequencing System (Illumina) using the NextSeq 500/550 High Output kit v2 (150 cycles, paired-end 75 bp).

### Annotation of genes

Gene models were predicted using the BRAKER2 pipeline (Brůna et al. 2021) using transcriptomic data as extrinsic evidence, yielding 22,338 gene models. Gene model completeness was assessed by predicting BUSCO genes (Simão et al. 2015). Functional annotations were lifted from the Maeda et al. (2018) gene annotation by intersecting genes with >50% matching sequence. Genes with transposon-related protein domains were removed (transposon|zinc_finger_bed_domain|ricesleeper|helicase-primase|helicase/primase|gag-pol|far1-related|ribonuclease_hi|ribonuclease_h|jockey|rve_super_family_integrase|transposase|transposable|helitron|pif1|zinc_finger_mym-type_protein_2|reverse_transcriptase). Remaining genes were classified based on predicted function and copy number. The top five protein families in expansion with known Pfam domains described in Tisserant et al. (2013) were named Class C (HCN). Genes with no known protein domain were classified as orphans (Class B). Class B genes that overlapped with repeats detected by RepeatModeler were further classified into HCN Class B genes and the others were considered LCN. All remaining genes were classified as core, LCN genes (Class A).

### Gene family phylogeny

Argonaute gene sequences from Maeda et al. (2018) were aligned using MUSCLE (version 3.8.31) (Edgar 2004) and a maximum-likelihood phylogenetic tree with 100 bootstraps was constructed using PhyML (version 3.3) (Guindon et al. 2010).

### Small RNA sequencing data processing

Raw sequencing reads were trimmed using cutadapt 1.9.1 (Martin 2011) using the parameters recommended in the NEXTflex Small RNA instructions. Read quality was assessed using FastQC v0.11.4 (https://www.bioinformatics.babraham.ac.uk/projects/fastqc/). Clean reads were aligned to a ribosomal RNA library made using SILVA database sequences for *R. irregularis*, and *Daucus_carota*_388.v2.0 using Bowtie v1.2.2 with the parameters -q -a -v 0 (Langmead et al. 2009). All reads perfectly matching rRNA or the carrot genome were filtered out. Remaining reads were aligned to the genome (parameters -q -k 500 -m 50 -v 1). sRNA profiles were plotted for collapsed reads using custom scripts. Reads from untreated, oxidized, and column-purified libraries were concatenated and clustered using Shortstack (parameters ‐‐dicermin 20 ‐‐dicermax27 ‐‐foldsize 300 ‐‐pad 200 ‐‐mincov 10.0rpmm ‐‐strand_cutoff 0.8) (Axtell 2013). sRNA counts were generated using featureCounts 1.5.0 using the Shortstack output and parameters -M ‐‐fraction -T 8 -F GTF -g ID -t nc_RNA (Liao et al. 2014).

### Poly(A)^+^ RNA sequencing data processing

RNA sequencing reads were filtered and trimmed by Novogene to remove low-quality reads (reads containing Qscore ≤5 in over 50% of bases, reads containing N > 10%), 5′ adapter 5′-AATGATACGGCGACCACCGAGATCTACACTCTTTCCCTACACGACGCTCTTCCGATCT-3′ and 3′ adapter 5′-GATCGGAAGAGCACACGTCTGAACTCCAGTCACATCACGATCTCGTATGCCGTCTTCTGCTTG-3′. Read quality was assessed using FastQC v0.11.4, and all samples showed a Phred score higher than 30. Reads were aligned to the unmasked *R. irregularis* DAOM197198 genome using STAR 2.5.4 (Dobin et al. 2013). For TE subfamily expression analyses, reads were aligned using the options ‐‐outFilterMultimapNmax 100 and ‐‐winAnchorMultimapNmax 100, and counts were generated using the TEtranscripts package (options ‐‐mode multi and ‐‐stranded no) with the gene annotation and a curated TE annotation file excluding simple repeats, unclassified repeats, low complexity repeats, satellites, rRNAs, snRNAs, and tRNAs (Jin et al. 2015). TE subfamilies with at least 100 normalized counts were considered expressed. For locus-level TE expression, counts were generated with featureCounts (subread package 2.0.1, ‐‐fraction parameter used) and only TEs of a length >100 bp and at least 1 RPKM in at least six samples were considered. For gene expression analysis, reads were mapped using STAR with the option ‐‐outFilterMultimapNmax 20, and counts were generated with featureCounts without the fraction option. Genes with at least two normalized counts in at least two samples were considered expressed. TE and gene counts were analyzed and plotted using the DESeq2, ggplot2 and pheatmap packages (version 1.0.12, https://rdrr.io/cran/pheatmap/; Love et al. 2014).

### ONT read processing and DNA methylation analysis

Genomic CpG methylation data was produced from Nanopore sequencing data with DeepSignal (0.1.8), called against model.CpG.R9.4_1D.human_hx1.bn17.sn360.v0.1.7+ using default parameters (Ni et al. 2019). From the DeepSignal output, data for symmetrical CG sites was merged and overlapped with TE or gene loci using BEDTools map -median.

### Software availability

Code used for analysis and visualization is available in Supplemental Code.

## Data access

All raw and processed sequencing data generated in this study have been submitted to the NCBI Gene Expression Omnibus (GEO; https://www.ncbi.nlm.nih.gov/geo/) under accession number GSE172187 and to the NCBI BioProject database (https://www.ncbi.nlm.nih.gov/bioproject/) under accession number PRJNA722386. Proteomics data are available via ProteomeXchange (http://www.proteomexchange.org) with identifier PXD025245.

## Supplementary Material

Supplemental Material
