# Supplementary material for: Transcriptional activity and epigenetic regulation of transposable elements in the symbiotic fungus Rhizophagus irregularis
Source: Genome Res. 2021 Dec;31(12):2290–302. doi: 10.1101/gr.275752.121 (PMC8647823; doi:10.1101/gr.275752.121)
Supplement: Supplemental Material [file supp_31_12_2290__DC1.html]

Transcriptional activity and epigenetic regulation of transposable elements in the symbiotic fungus Rhizophagus irregularis — Supplemental Material 

# Transcriptional activity and epigenetic regulation of transposable elements in the symbiotic fungus *Rhizophagus irregularis*

## Supplemental Material

- Supplemental\_Material.pdf
- Supplemental\_Code.zip
- Supplemental\_Table\_S1.xlsx
- Supplemental\_Table\_S2.xlsx
- Supplemental\_Table\_S3.xlsx
- Supplemental\_Table\_S4.xlsx
- Supplemental\_Table\_S5.xlsx
