# Supplementary material for: Transcriptional activity and epigenetic regulation of transposable elements in the symbiotic fungus Rhizophagus irregularis
Source: Genome Res. 2021 Dec;31(12):2290–302. doi: 10.1101/gr.275752.121 (PMC8647823; doi:10.1101/gr.275752.121)
Supplement: Supplemental Material [file supp_gr.275752.121_Supplemental_Material.pdf]

Supplemental Material

Transcriptional activity and epigenetic regulation of transposable elements in the symbiotic fungus *Rhizophagus irregularis*

Dallaire A*, [1, 2, 3], Manley BF[1, 2, 3], Wilkens M[4], Bista I[1, 2, 3], Quan C[5], Evangelisti E[5], Bradshaw CR[1], Ramakrishna NB[1, 2], Schornack S[5], Butter F[4], Paszkowski U*, #, [6], Miska EA*, #, [1, 2, 3].

Supplemental Tables

Supplemental Table S1. Differentially expressed TE subfamilies

Refer to Supplemental_Table_S1.xlsx

Supplemental Table S2. Expressed genes containing TEs or TE fragments

Refer to Supplemental_Table_S2.xlsx

Supplemental Table S3. Top 100 TEs closest to genes

Refer to Supplemental_Table_S3.xlsx

Supplemental Table S4. *R. irregularis* proteins detected by mass spectrometry

Refer to Supplemental_Table_S4.xlsx

Supplemental Table S5. Differentially expressed *R. irregularis* proteins

Refer to Supplemental_Table_S5.xlsx

Supplemental Table S6. Library sizes and statistics for RNA sequencing

Sample	Treatment	Library type	Sequenced	After filter 1 (rRNA)	After filter 2 (carrot)	Genome-mapped (final)	% mapped/ sequenced
0h_untreated_rep1	N/A	polyA+ RNA	24125647	N/A	N/A	23508030	97.44
0h_untreated_rep2	N/A		33011313	N/A	N/A	32179428	97.48
0h_untreated_rep3	N/A		26851644	N/A	N/A	26244797	97.74
0h_untreated_rep4	N/A		26369676	N/A	N/A	25633962	97.21
24h_untreated_rep1	N/A		27247136	N/A	N/A	26650424	97.81
24h_untreated_rep2	N/A		26387409	N/A	N/A	25656478	97.23
24h_untreated_rep3	N/A		22347632	N/A	N/A	21715194	97.17
24h_untreated_rep4	N/A		23269202	N/A	N/A	22606030	97.15
24h_exudate_rep1	N/A		26574357	N/A	N/A	25947202	97.64
24h_exudate_rep2	N/A		19888102	N/A	N/A	19436642	97.73
24h_exudate_rep3	N/A		25378856	N/A	N/A	24960105	98.35
24h_exudate_rep4	N/A		30133916	N/A	N/A	29510144	97.93
48h_untreated_rep1	N/A		23753967	N/A	N/A	23129238	97.37
48h_untreated_rep2	N/A		25007133	N/A	N/A	24336942	97.32
48h_untreated_rep3	N/A		24205949	N/A	N/A	23574174	97.39
48h_untreated_rep4	N/A		24370474	N/A	N/A	23727093	97.36
48h_exudate_rep1	N/A		21842200	N/A	N/A	21210960	97.11
48h_exudate_rep2	N/A		24461144	N/A	N/A	23893645	97.68
48h_exudate_rep3	N/A		23394216	N/A	N/A	22783627	97.39
48h_exudate_rep4	N/A		24240754	N/A	N/A	23697761	97.76
untreated_1_rep1	Untreated	Small RNA	8522362	3696746	3622866	3076779	36.1
untreated_1_rep2	Untreated		6185038	2495283	2452731	2155423	34.8
untreated_1_rep3	Untreated		3588142	1579416	1551398	1293706	36.1
untreated_1_rep4	Untreated		4801539	1963194	1934168	1702242	35.5
oxidised_1_rep1	NaIO4 oxidation		6822688	6461768	6402968	5169737	75.8
oxidised_1_rep2	NaIO4 oxidation		3949947	3550293	3475205	2476031	62.7
oxidised_1_rep3	NaIO4 oxidation		5614904	5366018	5290671	4018639	71.6
oxidised_1_rep4	NaIO4 oxidation		5061822	4601743	4541242	3603889	71.2
untreated_2_rep1	Untreated		13408373	2756044	2606783	2579412	19.2
untreated_2_rep2	Untreated		17095895	4012182	3875113	3821583	22.4
untreated_2_rep3	Untreated		19184550	4041787	4041787	3950481	20.6
column_2_rep1	TRaPR column		1224718	886033	884682	836745	68.3
column_2_rep2	TRaPR column		16027189	12188531	12168644	11494790	71.7
column_2_rep3	TRaPR column		12377676	9247436	9231218	8716589	70.4

Supplemental Figures

A

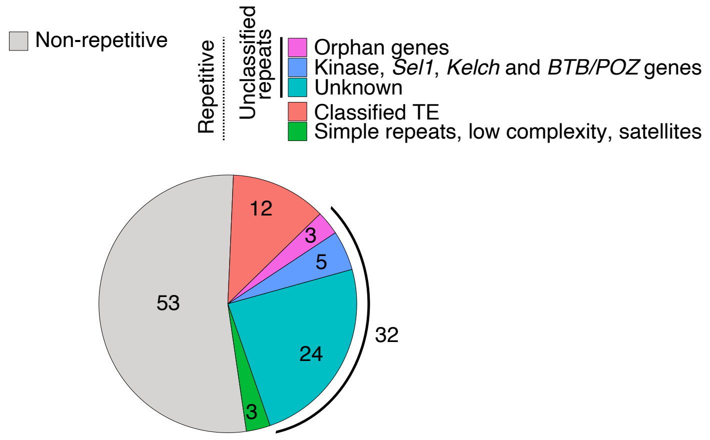

B

		Number	Length	%
**Retroelements**	**Total**	10902	8608092	5.75
	SINEs	13	430	0
	Penelope	210	16564	0.01
	LINEs	4836	5514118	3.68
	CRE/SLACS	6	259	0
	L2/CR1/Rex	385	29133	0.02
	R1/LOA/Jockey	921	2127706	1.42
	R2/R4/NeSL	209	163659	0.11
	RTE/Bov-B	137	73628	0.05
	L1/CIN4	503	366673	0.24
	LTR	6053	3093544	2.07
	BEL/Pao	197	17913	0.01
	Ty1/Copia	700	56798	0.04
	Gypsy/DIRS1	3442	2427958	1.62
	Retroviral	169	10001	0.01
**DNA transposons**	**Total**	20064	920978	6.15
	Hobo-Activator	4829	1861107	1.24
	Tc1-IS630-Pogo	3149	1111518	0.74
	En-Spm	2208	1487888	0.99
	PiggyBac	55	9013	0.01
	Tourist/Harbinger	239	53606	0.04
	Mirage, P-element, Transib	177	158418	0.11
	Helitron	954	516760	0.35
	Unclassified	144677	48379887	32.31
	Interspersed repeats		66190957	44.2
	Satellites	373	86118	0.06
	Simple repeats	67034	3045916	2.03
	Low complexity	15323	804954	0.54
**Bases masked : 47.2 %**				

C

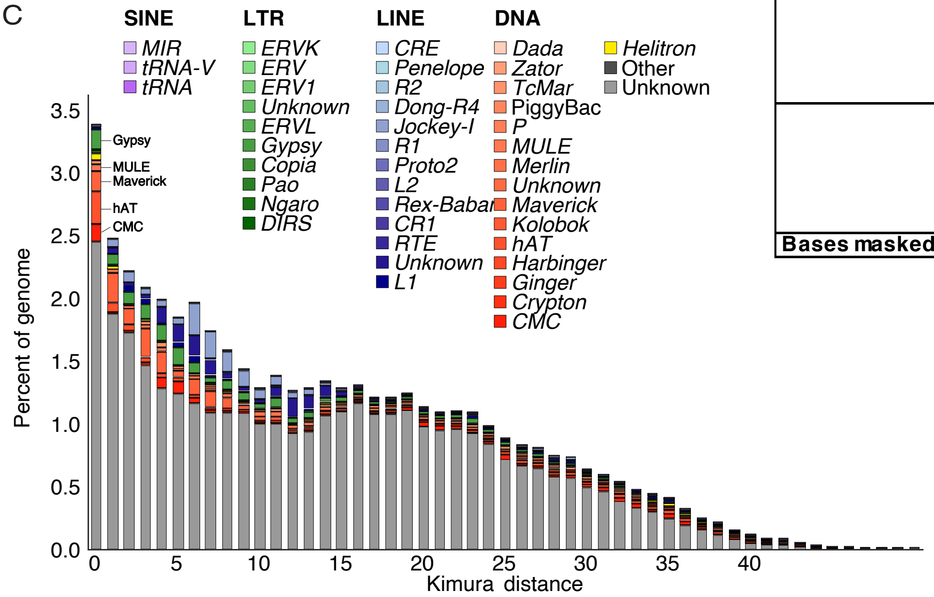

Supplemental Figure S1. Transposon and repeat annotation

A. Summary of genome sequence composition (%). **B**. The composition (%) of major TE families in *R. irregularis*, alongside associated length and copy number. **C**. Kimura distance-based copy divergence analyses of transposable elements in *R. irregularis*. Graph represents genome coverage (%) for each TE superfamily plotted against Kimura distances of TEs (CpG adjusted *K-value* from 0 to 50). TE copies with a low Kimura distance value have a low divergence from the consensus sequence and may correspond to recent replication events. Sequences with a higher Kimura distance value corresponded to older divergence. Data displayed is the same as data used to produce Figure 1A, and also includes unknown/unclassified elements.

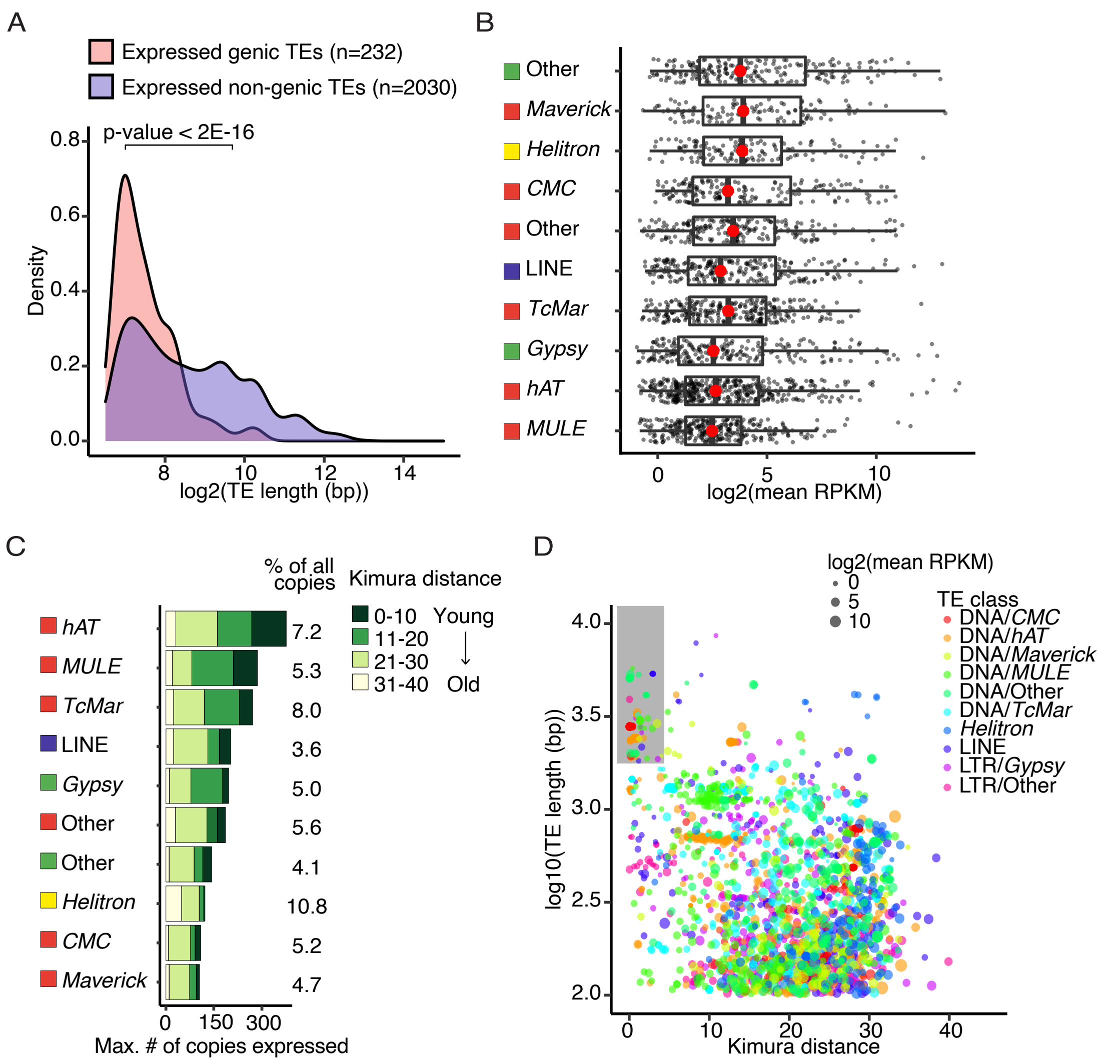

Supplemental Figure S2. Transposon expression at individual loci

A. Density plot depicting log-transformed TE length of genic (pink) and non-genic (purple) expressed individual TE loci. Expressed TEs were classified as 'genic' when found overlapping with the coding region of expressed genes. Expressed TEs that did not overlap with the coding regions of expressed genes were classified as non-genic. **B**. Non-genic TE expression levels, grouped by superfamily. Boxplots represent interquartile ranges and red dots represent the medians of log2 transformed mean RPKM across 20 samples (5 conditions, 4 replicates/condition). **C**. Number of expressed non-genic transposon copies of each super-family. Absolute TE numbers are displayed as percentage of expressed TEs compared to all TEs in the genome. Expressed TEs have been grouped into bins based on Kimura distance and hence relative age, represented using colour coding. **D**. Log-transformed length of all expressed TEs relative to divergence expressed as Kimura distance. Colour and point size indicate the TE class and log-transformed mean RPKM values respectively. Shaded area highlights TEs of >2kb length and <5% Kimura distance.

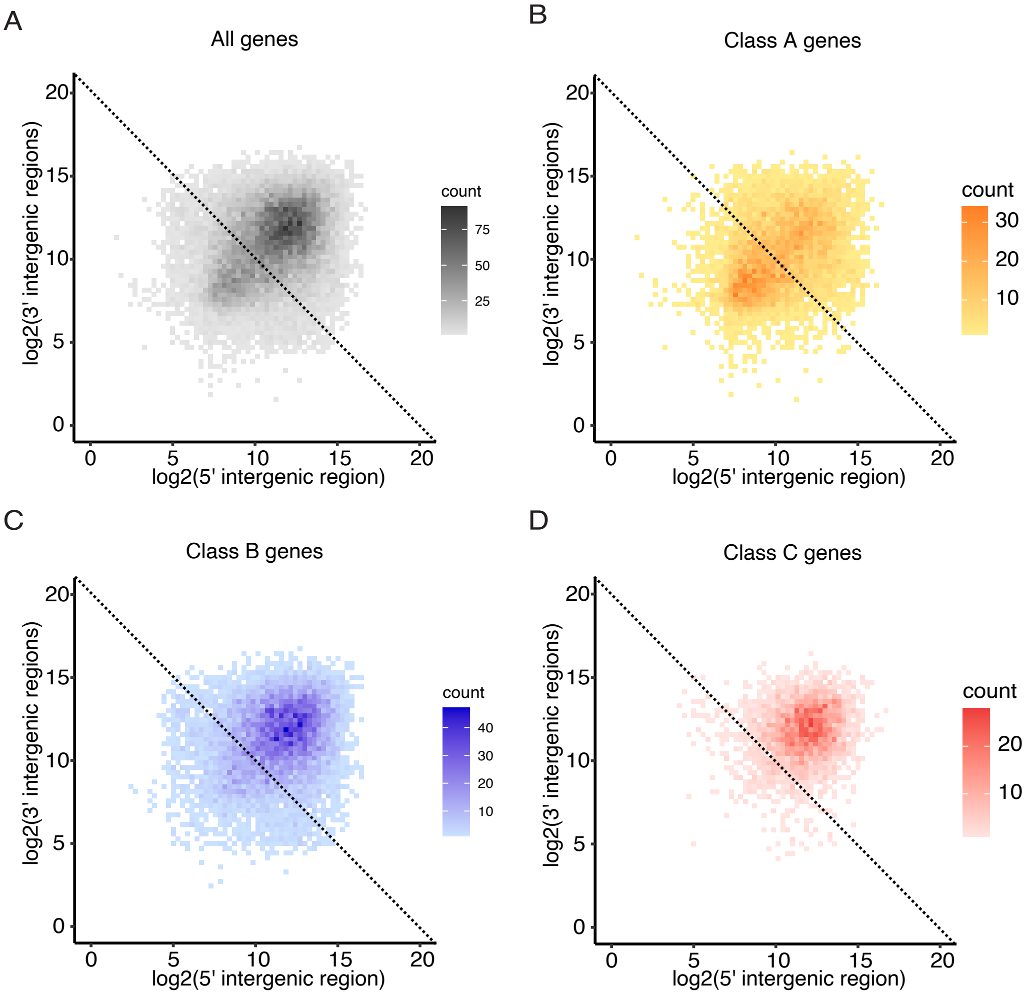

Supplemental Figure S3. Class-specific gene density in *R. irregularis*

Log-transformed intergenic disances upstream genes (5′) is plotted against log-tranformed intergenic distances downstream genes (3′) for all genes (**A**), class A (**B**), class B (**C**) and class C (**D**) genes.

A

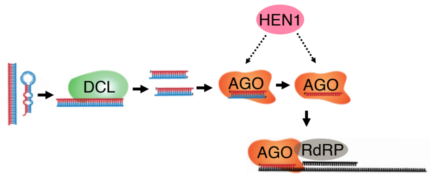

B

	Species	DCL	AGO	HEN1	RdRP
	Non-mycorrhizal	0-3	0-7	0-1	0-1
ECM	*L. bicolor*	2	6	0	6
ECM	*T. melanosporum*	2	3	0	2
Ericoid	*M. bicolor*	1	10	0	3
Ericoid	*P. ericae*	1	3	0	3
Orchid	*T. calospora*	1	9	0	3
AMF	***R. irregularis***	1	30	1	10
AMF	*R. clarus*	2	28	1	10
AMF	*G. cerebriforme*	2	23	1	10
Pathogen	*V. dahliae*	2	2	0	3
Pathogen	*B. cinerea*	1	2	0	2
Pathogen	*P. capsici*	1	5	0	1

C

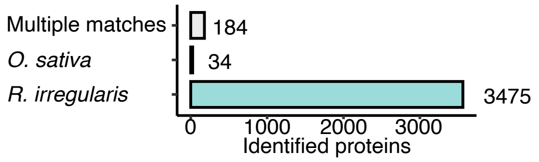

D

Uniprot ID	Protein	Log2(LFQ)	Coverage(%)
A0A2H5T0P1	DCL	23.3	7.7
A0A2H5R501	Argonaute binding protein	25.0	14.4
A0A2H5S4Q1	Argonaute binding protein	24.6	15.8
A0A2H5S8K1	Argonaute binding protein	25.1	25.8
A0A2H5R7T0	Argonaute	28.1	37.1
A0A2H5R7X8	Argonaute	28.8	25.4
A0A2H5R820	Argonaute	24.2	6.5
A0A2H5SS29	Argonaute	28.6	42.2
A0A2H5TEY9	Argonaute	24.9	16.8
A0A2H5TEZ5	Argonaute	24.4	19.5
A0A2H5TF38	Argonaute	26.8	31.9
A0A2H5UB68	Argonaute	24.4	11.8
A0A2H5UH87	Argonaute	30.9	59.9
U9URB1	Argonaute	29.8	53.4
U9SLB0	HRR1 helicase	25.1	7.3
A0A2H5TYD3	STC1	27.3	42.1
A0A2H5R5H6	HEN1	23.3	1.9

E

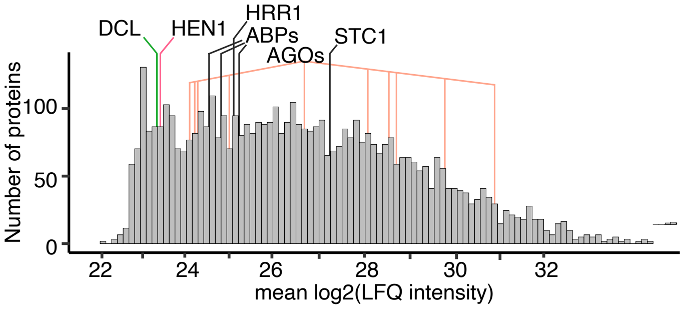

F

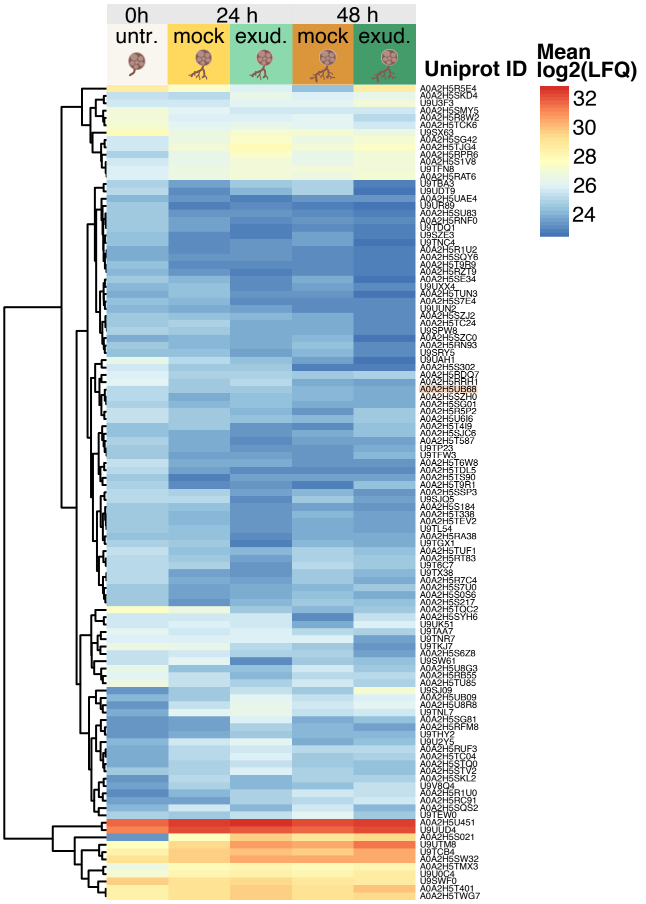

G

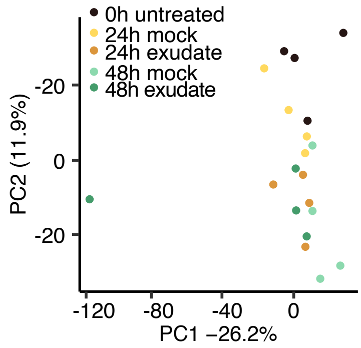

H

	Enriched GO terms		
	Term name	Term ID	Padj
↓24h exudate	Glycine import into mitochondrion	GO:1904983	4.998×10^{-2}
	Glycine transmembrane transporter	GO:0015187	4.979×10^{-2}
↓48h exudate	DNA replication initiation	GO:0006270	4.543×10^{-3}
	MCM complex	GO:0042555	1.218×10^{-3}

Supplemental Figure S4. RNAi-related genes in *R. irregularis* and their expression

A. Schematic representation of a typical RNAi pathway. Double-stranded RNA or hairpin RNAs are cleaved by the RNAse Dicer, generating sRNA duplexes. HEN1 methylates either the duplex or single-stranded sRNA loaded into AGO proteins. The sRNA-AGO complex then targets RNAs by base pairing. In some cases, RNA-dependent RNA polymerases (RdRPs) are recruited to facilitate silencing by using the target RNA as a template to generate more sRNA. **B**. Number of putative *DCL*, *AGO*, *HEN1* and *RdRP* genes found in genomes of species of mycorrhizal species: ectomycorrhizal (ECM), ericoid mycorrhiza, orchid mycorrhiza, arbuscular mycorrhiza (AMF) and pathogenic fungi capable of cross-kingdom sRNA transfer. **C**. Number of proteins identified by mass spectrometry matching to the proteomes of *R. irregularis*, *O. sativa* or both. **D**. Uniprot IDs, label-free quantitation (mean log-transformed LFQ intensities) and % unique coverage of RNAi pathway genes detected by proteomics. **E**. Distribution of LFQ intensity of proteins detected by mass spectrometry. Values for RNAi pathway proteins are indicated. **F**. Heatmap and hierarchical clustering of mean log-transformed LFQ intensities of differentially expressed *R. irregularis* proteins ($|\Delta\ \log2(LFQ)| > 1.1$; $\log10(Padj) > 1.1$) at 24h or 48h post-treatment with mock (Hoagland's; nutrient condition) or exudate (rice root exudates) treatment relative to control conditions (0h; no treatment). ID marked in orange is an Argonaute protein. **G**. Principal component analysis of protein expression across all replicates and treatments, 0h control, 24h mock and rice exudate treatments, and 48h mock and rice exudate treatment (20 samples total, at 4 replicates per treatment). **H**. Functional enrichment analysis of differentially expressed proteins using g:Profiler (Raudvere et al. 2019).
